# Supplementary material for: Entangled polymer dynamics beyond reptation
Source: Nat Commun. 2018 Nov 30;9:5098. doi: 10.1038/s41467-018-07546-7 (PMC6269522; doi:10.1038/s41467-018-07546-7)
Supplement: Supplementary file 1 — Supplementary Information [file 41467_2018_7546_MOESM1_ESM.docx]

Supplementary Information for

Entangled polymer dynamics beyond reptation

Maram Abadi, Maged F. Serag, Satoshi Habuchi*

King Abdullah University of Science and Technology (KAUST), Biological and Environmental Sciences and Engineering Division, Thuwal 23955-6900, Saudi Arabia

**Contents**

Supplementary Figures 1 to 4

**Supplementary Figures**



**Supplementary Figure 1. Long term mechanical stability of the microscope along axial direction.** The z-axis positions of TetraSpeck fluorescent nanospheres (diameter of 100 nm) deposited on a cleaned cover slip were determined by 3D astigmatism-based fluorescence localization microscopy over 15 min. This data confirmed that the stage drift along the z-axis during 15 min image acquisition is less that ±20 nm.


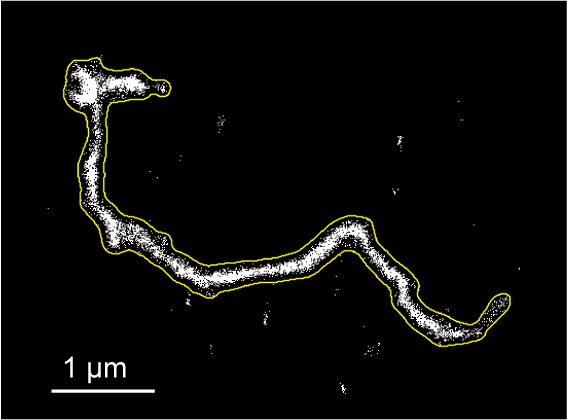


**Supplementary Figure 2. Identification of the localized spots obtained from the Cy5-labeled DNA molecule.** White dots show localized spots. The solid line is the region determined by the Moore-Neighbor tracing algorithm. The localizations included in this region are regarded as the spots obtained from the DNA molecule.


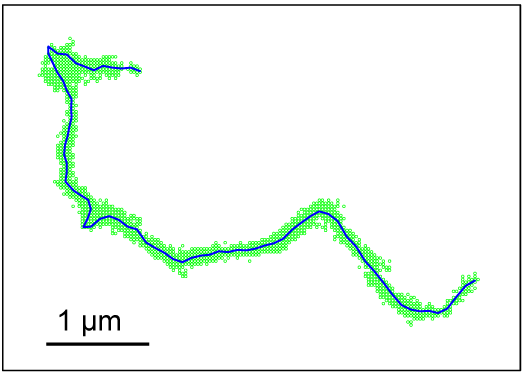


**Supplementary Figure 3.** **Local direction of the contour of the Cy5-labeled DNA molecule determined by piecewise linear mapping.** The dots show the localized spots. The solid line shows a roughly-estimated contour of the molecule obtained by the fitting of each segment to piecewise linear function.


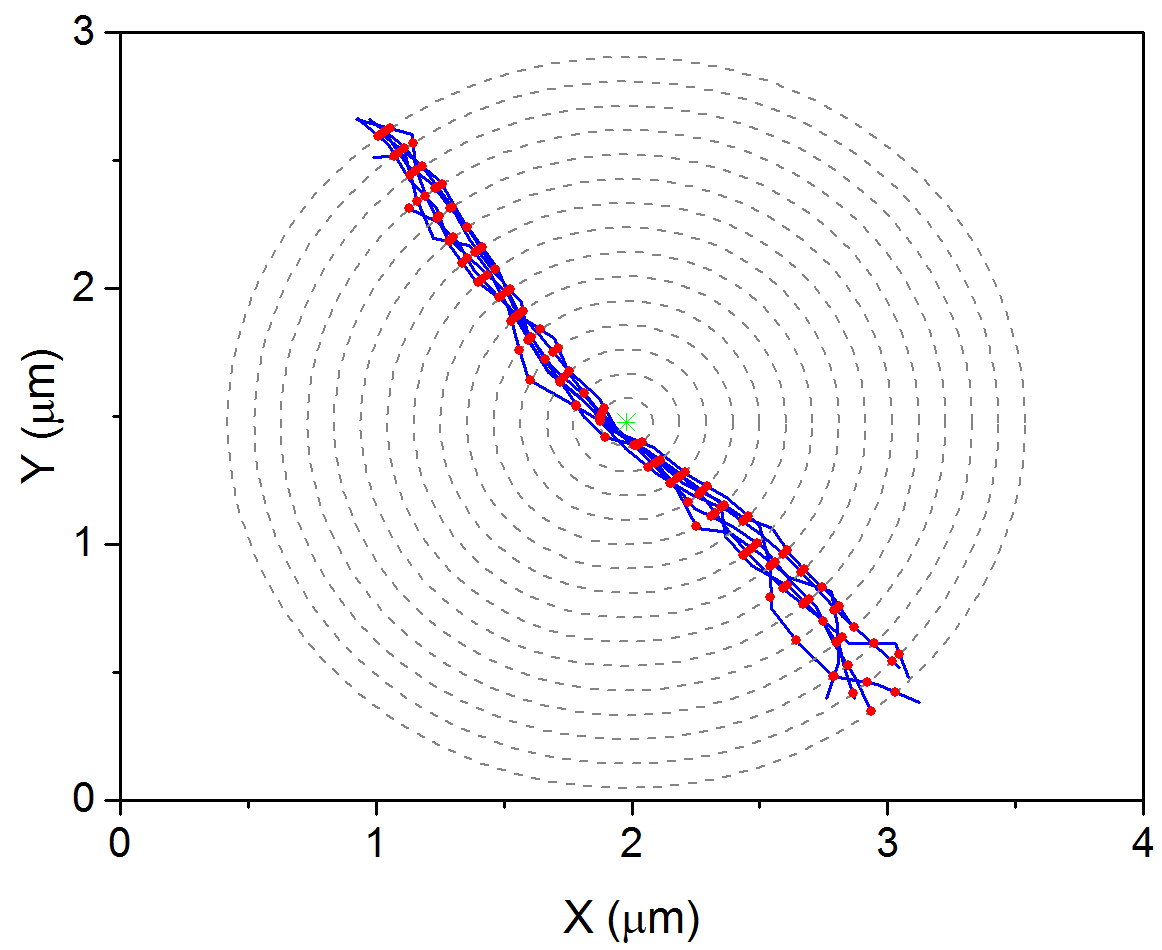


**Supplementary Figure 4.** **Quantification of the local chain displacements occurring during the time scale of capturing consecutive super-resolution images.** Centre of masses (CMs) obtained from consecutive frames are spatially aligned (blue lines). The position-dependent displacement at each segment was determined by measuring the largest displacement between the local chain positions (red dots) obtained from the segments located at the same distance from the CM (dashed lines).
